# Unilateral Sensorineural Hearing Loss in Children Associated With Sjögren's Syndrome

**DOI:** 10.7759/cureus.18832

**Published:** 2021-10-17

**Authors:** Yuko Okawa, Kazuo Okanari, Naoki Hirano, Toshiaki Kawano, Shinya Nishio, Shinichi Usami, Tomoki Maeda, Kenji Ihara

**Affiliations:** 1 Pediatrics, Oita University School of Medicine, Yufu, JPN; 2 Otolaryngology-Head and Neck Surgery, Oita University School of Medicine, Yufu, JPN; 3 Hearing Implant Sciences, Shinshu University School of Medicine, Matsumoto, JPN

**Keywords:** autoimmune mediated inner ear disease, high-frequency hearing loss, sensorineural hearing loss, deafness in childhood, sjögren's syndrome

## Abstract

The occurrence of unilateral sensorineural hearing loss (SNHL) during school age is relatively rare and accounts for approximately 6% of all deafness in childhood. We present two cases involving children who were diagnosed with SNHL associated with Sjögren's syndrome (SS). Case 1: An eight-year-old girl with an approximately two-year clinical history of gradual hearing loss was diagnosed with SNHL associated with SS based on histological findings of inflammation in the salivary glands and the presence of serum anti-Sjögren's syndrome-A antibody. Case 2: An eight-year-old boy with acute idiopathic thrombocytopenic purpura in whom unilateral hearing loss, which was not associated with any problems in daily life, was detected during hospitalization and who was finally diagnosed with SNHL and SS. Steroid treatment was ineffective for both patients. The previously unrecognized combination of SNHL with SS should be considered in the diagnosis of unilateral SNHL, even in children.

## Introduction

Congenital sensorineural hearing loss (SNHL) is one of the most common congenital disorders [1], with a prevalence of one to two per thousand live births; its major etiologies include genetic causes (approximately two-thirds), cytomegalovirus (CMV) infection (21%), and other environmental causes (14%) [2]. Most patients with SNHL are identified by newborn hearing screening with an automated auditory brainstem response (AABR) examination during the neonatal period or by routine health check-ups for preschool children. In contrast, it is relatively uncommon to diagnose acute hearing loss in patients with SNHL during school age (6-12 years), accounting for approximately 6% of all children with hearing difficulty. The etiology of SNHL in older children differs from that of younger children, as structural abnormalities account for approximately half (44%) of SNHL cases, while 37% have an unknown origin [1]. 

We herein report two cases of children with unilateral SNHL associated with Sjögren's syndrome (SS).

## Case presentation

Case 1

**Figure 1:**
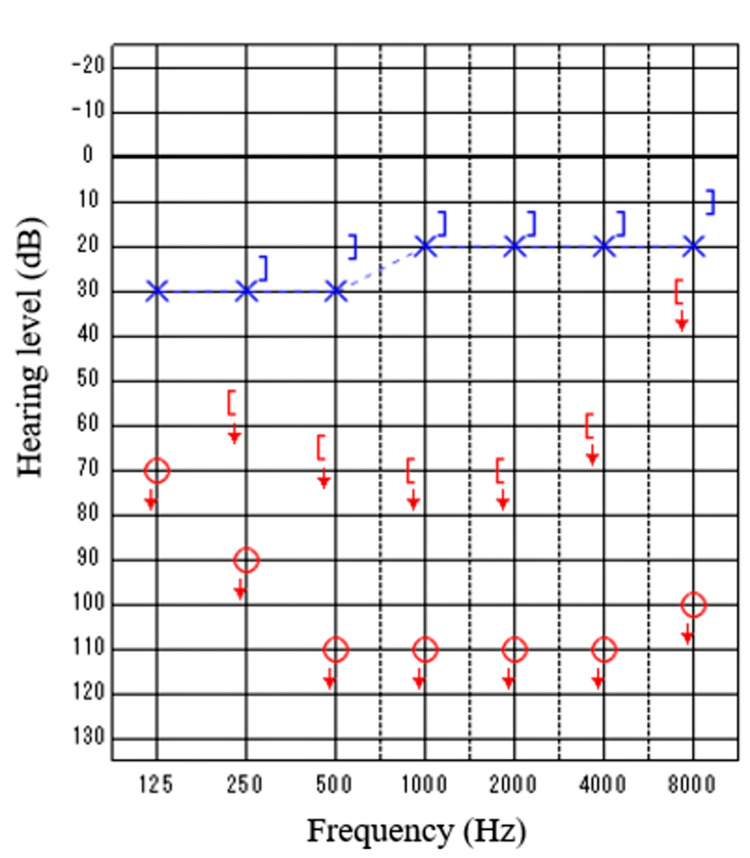
Hearing tests of Case 1: Pure tone audiometry Air conduction hearing levels in the right ear (circles) and left ear (cross marks), and bone conduction hearing levels in the right ear ( [ ) and left ear ( ] ). No response, even at the maximum volume that can be output from the machine indicates the scale-out of the threshold (arrows).

**Figure 2:**
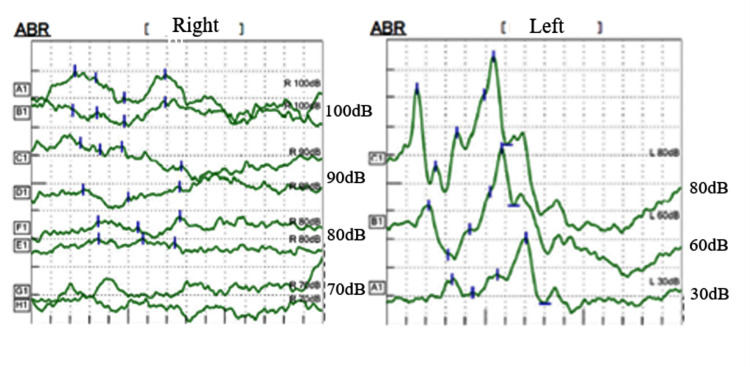
Hearing tests of Case 1: ABR The responses at 30, 60, and 80 dB are shown in the left ear. The right ear was examined twice at 80, 90, and 100 dB and all waveforms were poorly recorded, indicating no response. ABR, auditory brainstem response.

**Figure 3:**
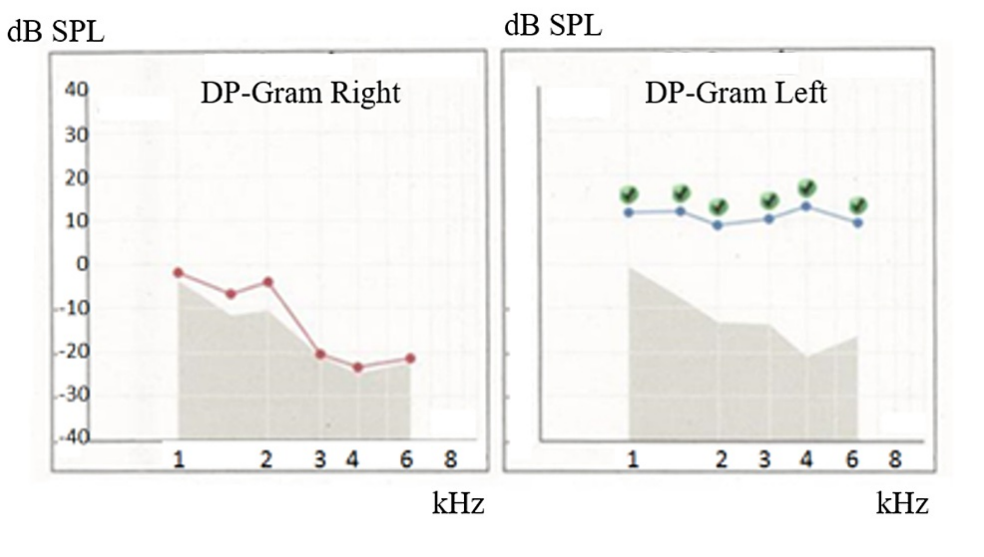
Hearing tests of Case 1: DPOAEs The DP-gram was drawn from the DP levels. Green round dots show each response at stimulus SPL. DPOAEs in the right ear were absent at any frequency level. DP-gram, distortion product-gram; DP, distortion product; SPL, sound pressure level; DPOAE, distortion product otoacoustic emission.

**Figure 4:**
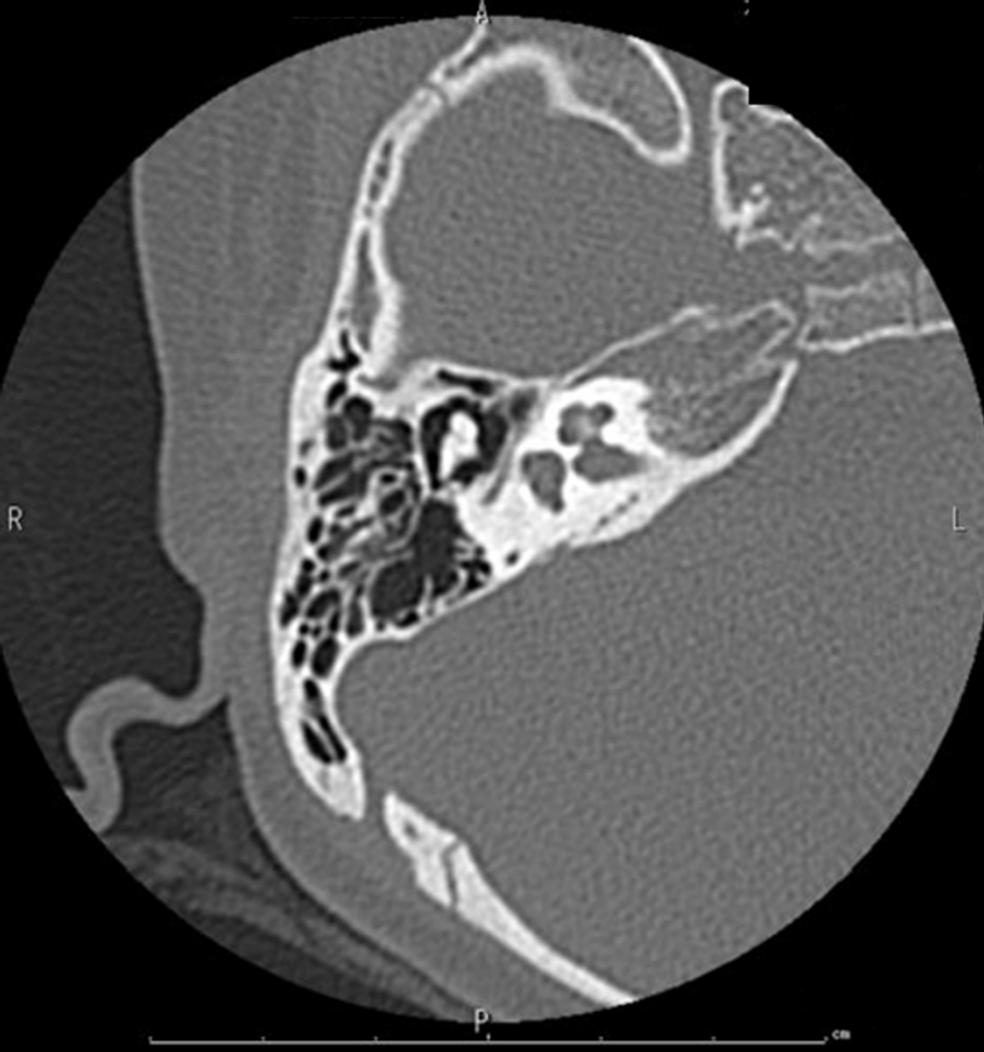
Imaging of Case 1: temporal bone CT (right) Affected side. CT, computed tomography.

**Figure 5:**
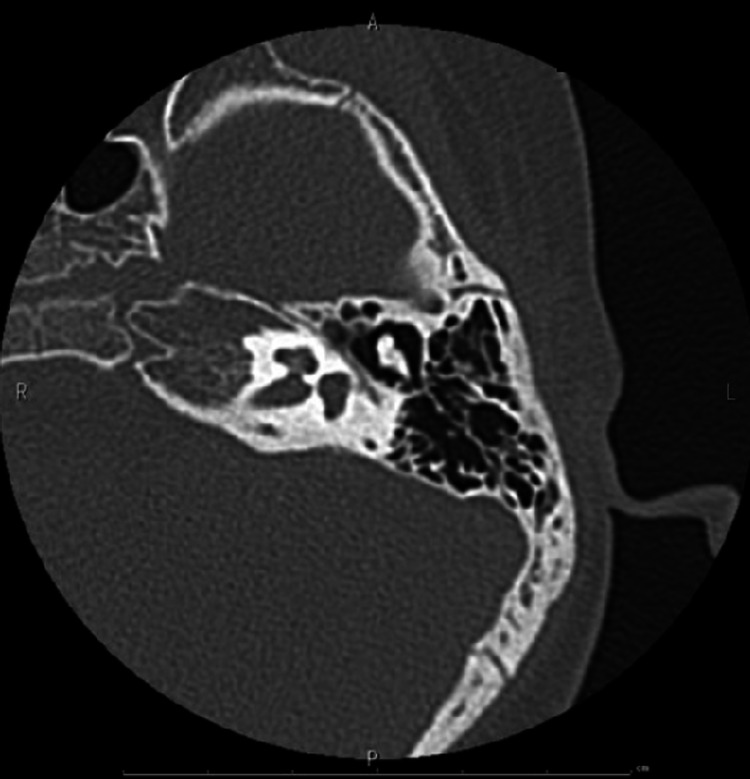
Imaging of Case 1: temporal bone CT (left) Healthy side. CT, computed tomography.

**Figure 6:**
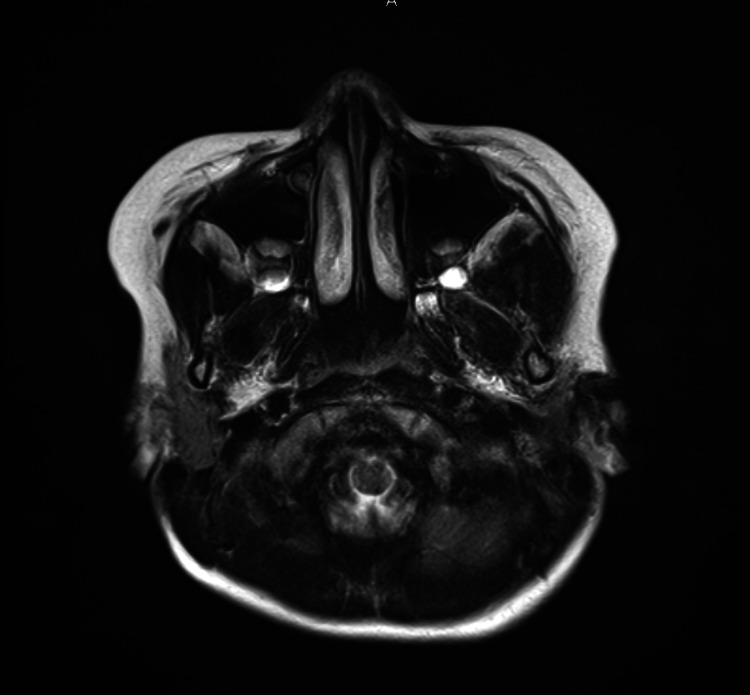
Imaging of Case 1: temporal MRI (T2-weighted image) MRI, magnetic resonance imaging.

The patient was an eight-year-old girl with hearing loss in her right ear. She passed hearing screening (an AABR examination) at birth. At approximately six years of age, she noticed difficulty in daily communication with others. A pure tone audiometry test showed profound hearing loss in the right ear and normal hearing in the left ear (Figure 1). In an auditory brainstem response (ABR) examination of the right ear, all I-V waves were poorly identified (Figure 2). The distortion product otoacoustic emissions (DPOAEs) in the right ear were absent, indicating severe unilateral SNHL (Figure 3). Temporal bone computed tomography (CT) and magnetic resonance imaging (MRI) showed no structural abnormalities in either of the inner ears (Figures 4-6). Quantitative polymerase chain reaction (qPCR) for the detection of CMV deoxyribonucleic acid (DNA) in stored dried umbilical cord was negative [3]. An additional serum analysis to detect the etiology of SNHL revealed the following: anti-CMV immunoglobulin (Ig)-G antibody, 236 AU/mL (cutoff, <6); anti-CMV IgM (cutoff, <0.85) and anti-mumps IgM antibodies (cutoff, <0.8), below the limit of detection; anti-Sjögren's syndrome-A antibody, 135.1 U/mL (cutoff, <10). Based on the findings of a Saxon test, which showed the decreased production of saliva, and the pathological findings of salivary gland biopsy presenting numerous plasma cell and lymphocyte infiltrations around the salivary gland ducts, we diagnosed the patient with SS. Standard treatment for idiopathic sudden deafness with prednisolone (1 mg/kg/day for 10 days, tapered off for four days) showed no improvement in the hearing ability of either ear evaluated by pure tone audiometry, ABR, and DPOAEs at six months.

 

Case 2

**Figure 7:**
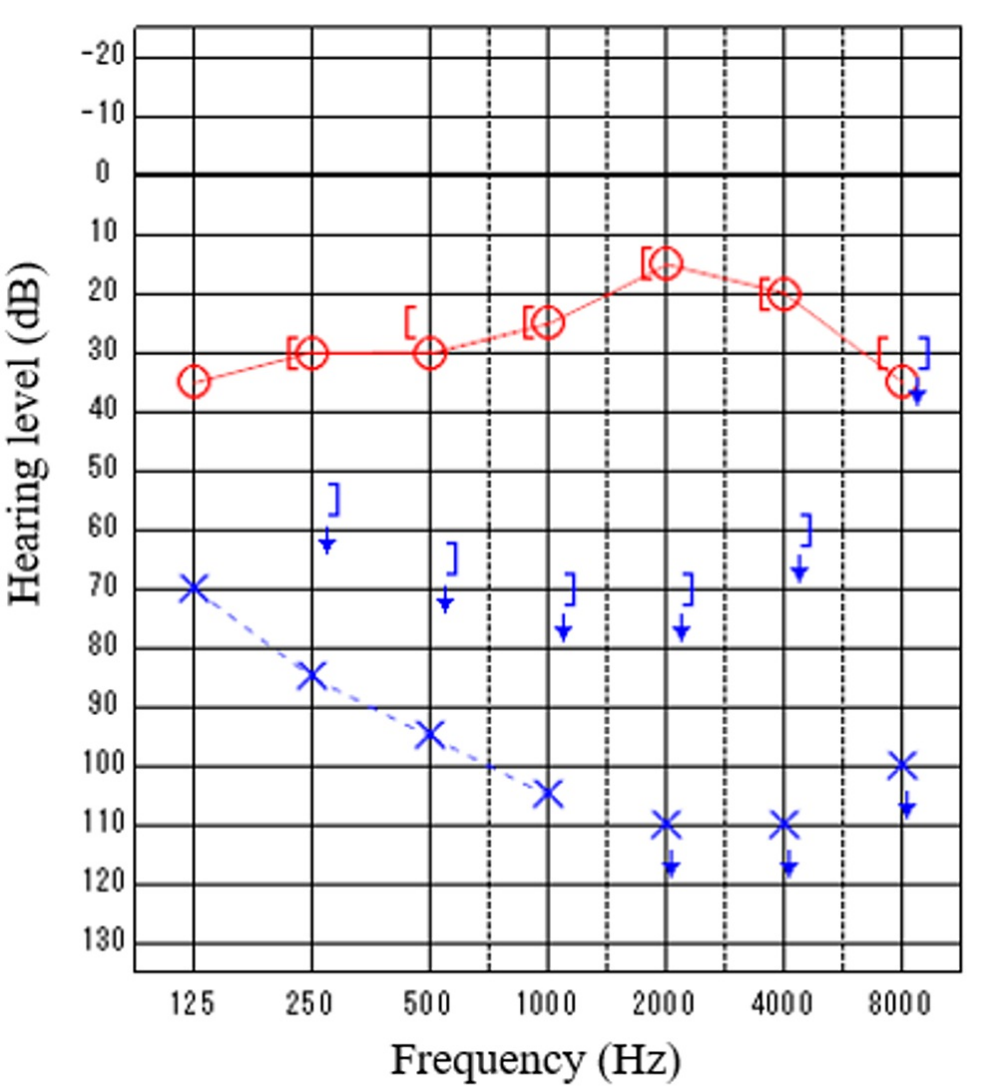
Hearing tests of Case 2: Pure tone audiometry

**Figure 8:**
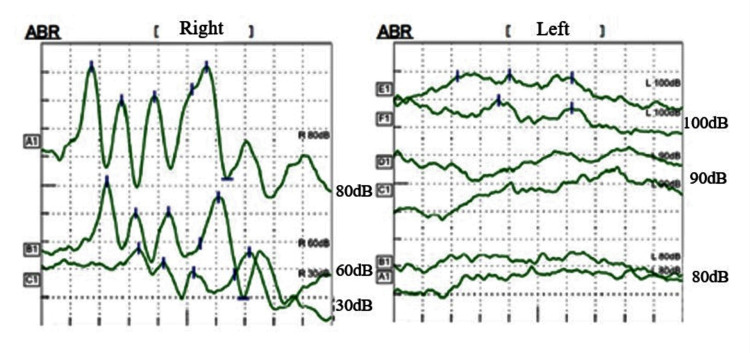
Hearing tests of Case 2: ABR ABR, auditory brainstem response.

**Figure 9:**
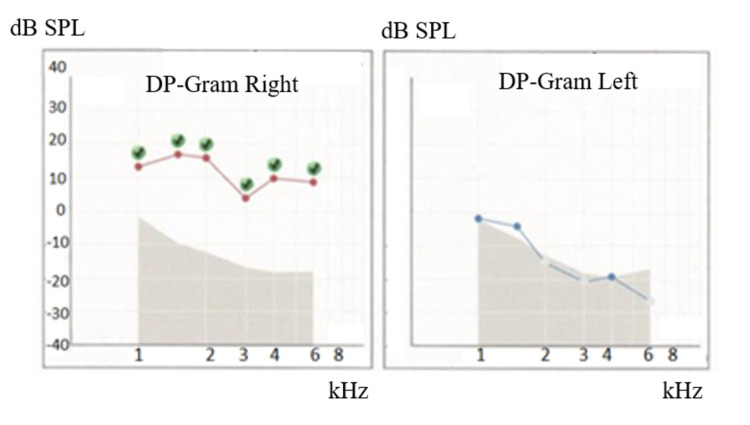
Hearing tests of Case 2: DPOAEs DP-gram, distortion product-gram; DP, distortion product; SPL, sound pressure level; DPOAE, distortion product otoacoustic emission.

**Figure 10:**
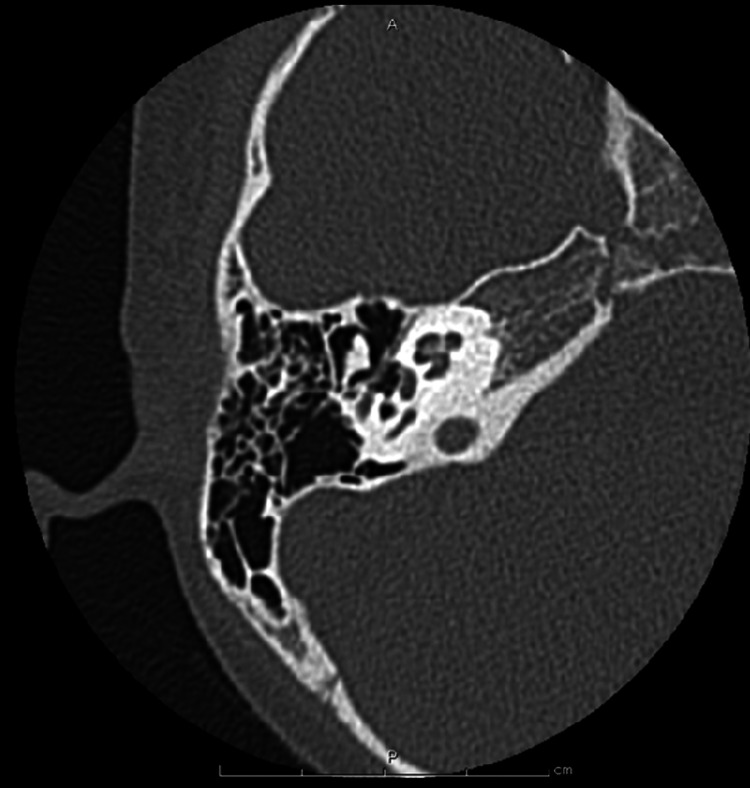
Imaging of Case 2: temporal bone CT (right) Healthy side. CT, computed tomography.

**Figure 11:**
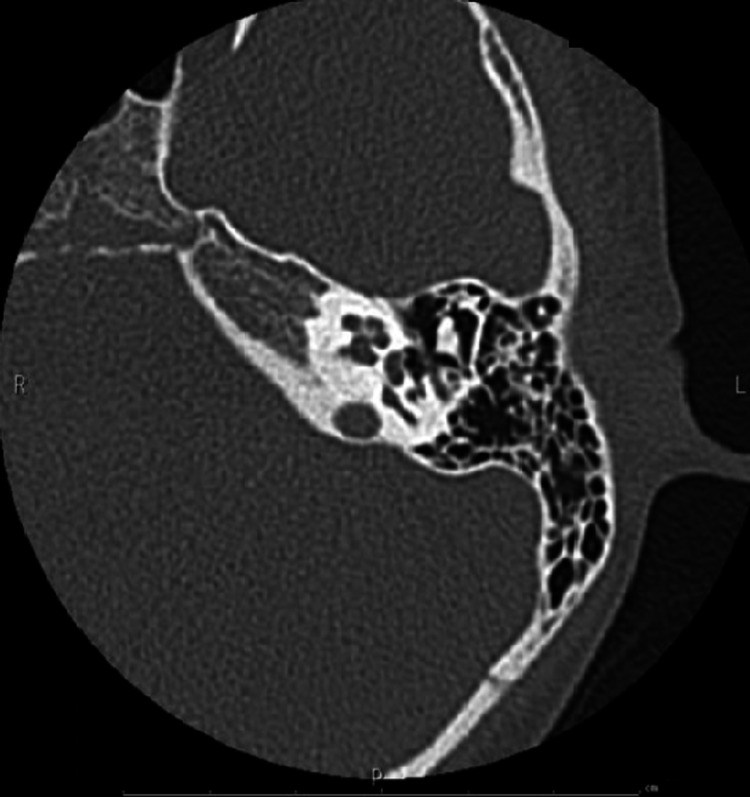
Imaging of Case 2: temporal bone CT (left) Affected side. CT, computed tomography.

**Figure 12:**
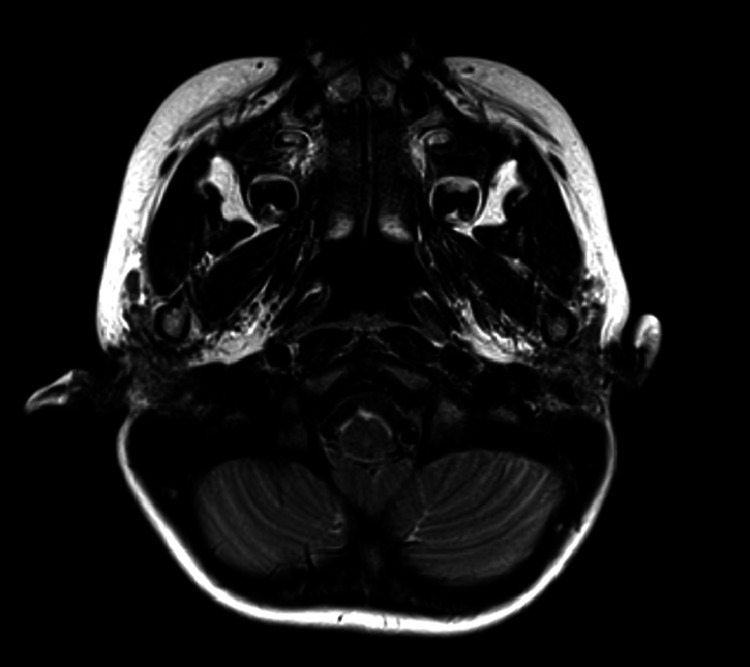
Imaging of Case 2: temporal MRI (T2-weighted image) MRI, magnetic resonance imaging.

An eight-year-old boy presented to our hospital due to the sudden occurrence of subcutaneous hemorrhage and bruises on his extremities. He was diagnosed with acute immune thrombocytopenia (ITP) based on isolated thrombocytopenia (<50,000/μL) with the presence of platelet-associated IgG at 66 ng/10^7^ cells (cutoff level, <46). In a bone marrow examination, normal proliferation and differentiation of lymphoid and myeloid lineage cells with increased megakaryocytes were observed, a typical finding of ITP. He suffered from acute gastroenteritis with remarkable thrombocytopenia and was admitted to our hospital for the management and treatment of gastroenteritis and thrombocytopenia. During admission, he noticed hearing difficulty in his left ear. Pure tone audiometry demonstrated profound hearing loss in the left ear from 2,000 to 8,000 Hz (Figure 7). In ABR examination of the left ear, all I-V waves were poorly identified (Figure 8). DPOAEs in the left ear were absent, indicating severe unilateral SNHL (Figure 9). Temporal bone CT and MRI showed no structural abnormalities in the inner ears (Figures 10-12). His serum test findings were as follows: anti-CMV IgG antibody, 106 AU/mL (cutoff, <6); anti-Sjögren's syndrome-B antibody, 13.2 U/mL (cutoff, <10); his anti-CMV IgM and anti-mumps IgM antibody levels were below the limit of detection. We checked his mother and child health handbook, and found that he had not undergone newborn hearing screening using AABR test at birth. His stored dried umbilical cord was examined by qPCR for CMV and was found to be negative. A fluorescein test and Saxon test were both positive. The histopathological examination of a salivary gland biopsy specimen revealed the infiltration of lymphocyte and plasma cells around the salivary gland duct, as typical findings of SS.

On the 12th day, he was treated with intravenous immunoglobulin (1 g/kg) for thrombocytopenia, showing a good response with a platelet count increase from 7,000 to 177,000 within three days. Steroid treatment was started (prednisolone, 1 mg/kg/day) on the 19th day of admission, and tapered off after 18 days. His platelet level successfully remained at >50,000/μL; however, no remarkable change in hearing activity in his left ear was observed by ABR or DPOAEs in the three months after the treatment.

## Discussion

These two children, in whom unilateral hearing loss was noticed at school age, were diagnosed with unilateral SNHL associated with SS. The affected region causing hearing loss was identified as intra-cochlea based on a DPOAE test and pure tone audiometry. Because the condition was not responsive to steroid and immunoglobulin therapy, it was hypothesized that inflammation triggered by an autoimmune reaction causing atrophy and necrosis had irreversibly damaged the cochlea. We unexpectedly found the co-existence of SS in both patients, and it was hypothesized that the autoimmune reaction induced by SS might have, at least in part, played a role in the irreversible damage of the cochlea. The pathophysiological rationale for our speculation is as follows. (i) A recent report demonstrated that SS patients diagnosed in adulthood showed a very high prevalence (95.2%) of SNHL in high-frequency ranges [4]. Both cases showed similar damage in the high-frequency ranges. (ii) Examination of the stored umbilical cord tissues was negative for CMV, which is the most common cause of unilateral SNHL in children. (iii) Inner ear malformation, which is the most frequent etiology of unilateral SNHL in school-age children, was not detected by CT or MRI. (iv) The co-occurrence of ITP with SS or with acute hearing loss is not rare even in children as shown in the SS guidelines or the previous reports, suggesting the presence of autoimmunity [5].

The pathophysiology of the inner ear involvement in SS is still unknown. Calzada and Hatzopoulos have reported vacuolization and thickening of the basement membrane as well as deposits of immune complexes in the stria vascularis, producing arterial microvasculature ischemia of the basal turn of the cochlea, where high frequencies are detected [4]. It is presumed that cochlear macrophages present in the cochlear ganglion and stria vascularis mobilize inflammatory macrophages in autoimmune inflammatory inner ear disease. As a result, inflammation of the inner ear blood vessels causes atrophy of the stria vascularis and reduces blood flow. Vascular ischemia causes necrosis or fibrosis of the hair cells and the cochlear ganglion [6]. Similar to age-related hearing loss, cochlear hair cells are damaged and shed from the cochlear entrance, and necrotic hair cells do not regenerate. Since the inner ear is a small tissue, the biopsy is difficult and cannot be proved, but as inflammation progresses, necrosis and shedding of hair cells may lead to irreversible hearing loss. In adults, there are several reports of SS with hearing loss (Tables 1, 2) [4,7-14]. It has been estimated that auto-antigens released from one side of the damaged inner ear could trigger an autoimmune reaction in the other side of the inner ear, causing hearing loss in both ears with a variety of severity [15].

**Table 1:** Summary of the past literature on hearing loss with Sjögren’s syndrome: Case report PSL, prednisolone; mPSL, methylprednisolone; ND, not documented.

Reference [No.]	Age/sex	Bilateral/unilateral	Frequency band (dB)	Treatment (dose)	Clinical course
Almeida RS, et al. [7]	65/Female	Unilateral	8,000 Hz (70 dB)	PSL (1 mg/kg/day)	Effective, with recurrence
Kadosono O, et al. [8]	46/Female	Bilateral	ND	mPSL (500 mg/day for 3 days)	Effective
Kim KS and Kim HS [9]	62/Female	Unilateral	ND	mPSL (250 mg/day for 5 days)	Effective

**Table 2:** Summary of the past literature on hearing loss with Sjögren’s syndrome: Case study ND, not documented.

Reference [No.]	Number of SjS (F/M)	SNHL (%)	Frequency band of SNHL (Hz)	Bilateral/unilateral (number)
González JLT, et al. [4]	63 (60/3)	95.2	10,000-16,000	Bilateral (all)
Gündüz B, et al. [10]	36 (36/0)	52.77	9,000-12,500 Hz	ND
Galarza-Delgado DA, et al. [11]	60 (60/0)	60, 70, 100	500-3,000 Hz, 4,000-8,000 Hz, 10,000-16,000 Hz	ND
Agrup C. [12]	ND	36	ND	ND
Ziavra N, et al. [13]	45 (45/0)	22.5	3,000-8,000 Hz (8 patients)	Bilateral (4), unilateral (5)
Tumiati B, et al. [14]	30 (30/0)	46	2,000-8,000 Hz (12 patients)	Bilateral (10), unilateral (4)

In contrast, there have been no reports of hearing loss in pediatric SS, and the hearing loss of patients was not responsive to steroid treatment. Thus, it might be possible that hearing loss and SS were simply coincidental. It is possible that congenital CMV infection may underlie the cause of deafness because the sensitivity of the CMV test is not 100%; furthermore, a trivial congenital malformation in the inner ear might have been undetectable by MRI or CT.

## Conclusions

The previously unrecognized combination of SNHL with SS should be considered in the diagnosis of unilateral SNHL, even in children. Further study, with a greater number of cases and in vitro studies to help determine the actual reaction cascade, would be needed to conclude that SS can directly cause hearing loss by an immune reaction in the inner ear cells, and to determine whether an early diagnosis and steroid treatment can change the hearing prognosis of patients with SNHL with SS.
